# Methylation analysis of histone H4K12ac-associated promoters in sperm of healthy donors and subfertile patients

**DOI:** 10.1186/s13148-015-0058-4

**Published:** 2015-03-19

**Authors:** Markus Vieweg, Katerina Dvorakova-Hortova, Barbora Dudkova, Przemyslaw Waliszewski, Marie Otte, Berthold Oels, Amir Hajimohammad, Heiko Turley, Martin Schorsch, Hans-Christian Schuppe, Wolfgang Weidner, Klaus Steger, Agnieszka Paradowska-Dogan

**Affiliations:** Section Molecular Andrology, Biomedical Research Center Seltersberg, Justus Liebig University of Giessen, 35392 Giessen, Germany; Laboratory of Reproductive Biology, Institute of Biotechnology AS CR, v.v.i., Videnska 1083, 14220 Prague 4, Czech Republic; Biocev Group, Department of Zoology, Faculty of Science, Charles University in Prague, 12844 Prague, Czech Republic; Department of Urology, Pediatric Urology and Andrology, Justus Liebieg University of Giessen, 35392 Giessen, Germany; Fertility Center, 35578 Wetzlar, Germany; Fertility Center, 65189 Wiesbaden, Germany

**Keywords:** H4K12ac in spermatozoa, μChIP, Promoter methylation, Pyrosequencing, Subfertility

## Abstract

**Background:**

Histone to protamine exchange and the hyperacetylation of the remaining histones are hallmarks of spermiogenesis. Acetylation of histone H4 at lysine 12 (H4K12ac) was observed prior to full decondensation of sperm chromatin after fertilization suggesting an important role for the regulation of gene expression in early embryogenesis. Similarly, DNA methylation may contribute to gene silencing of several developmentally important genes. Following the identification of H4K12ac-binding promoters in sperm of fertile and subfertile patients, we aimed to investigate whether the depletion of histone-binding is associated with aberrant DNA methylation in sperm of subfertile men. Furthermore, we monitored the transmission of H4K12ac, 5-methylcytosine (5mC) and 5-hydroxymethylcytosine (5hmC) from the paternal chromatin to the embryo applying mouse *in vitro* fertilization and immunofluorescence.

**Results:**

Chromatin immunoprecipitation (ChIP) with anti-H4K12ac antibody was performed with chromatin isolated from spermatozoa of subfertile patients with impaired sperm chromatin condensation assessed by aniline blue staining. Fertile donors were used as control. DNA methylation analysis of selected H4K12ac-interacting promoters in spermatozoa was performed by pyrosequencing.

Depletion of binding sites for H4K12ac was observed within the following developmentally important promoters: AFF4, EP300, LRP5, RUVBL1, USP9X, NCOA6, NSD1, and POU2F1. We found 5% to 10% hypomethylation within CpG islands of selected promoters in the sperm of fertile donors, and it was not significantly altered in the subfertile group. Our results demonstrate that the H4K12ac depletion in selected developmentally important promoters of subfertile patients was not accompanied by a change of DNA methylation.

Using a murine model, immunofluorescence revealed that H4K12ac co-localize with 5mC in the sperm nucleus. During fertilization, when the pronuclei are formed, the paternal pronucleus exhibits a strong acetylation signal on H4K12, while in the maternal pronucleus, there is a permanent increase of H4K12ac until pronuclei fusion. Simultaneously, there is an increase of the 5hmC signal and a decrease of the 5mC signal.

**Conclusions:**

We suggest that aberrant histone acetylation within developmentally important gene promoters in subfertile men, but not DNA methylation, may reflect insufficient sperm chromatin compaction affecting the transfer of epigenetic marks to the oocyte.

**Electronic supplementary material:**

The online version of this article (doi:10.1186/s13148-015-0058-4) contains supplementary material, which is available to authorized users.

## Background

The classic definition of infertility for both, men and women, states a disability to conceive within 12 months, despite frequent unprotected sexual intercourse. About 15% of couples have difficulties to become pregnant, and in approximately 30% of these couples, it is only the man who is diagnosed with reproductive problems [1]. There is a growing population of men with infertility disorders, having a decreased number of sperm cells in the ejaculate, as assessed by sperm analysis (oligozoospermia), or no sperm cells present in the ejaculate (azoospermia) [2]. Over 80% of men suffering with infertility have a low sperm concentration associated with decreased sperm motility and a normal sperm morphology (asthenozoospermia). Others may have decreased sperm motility and also abnormal sperm morphology (teratozoospermia). Besides severe forms of male infertility, which is referred to as azoospermia, reduced fertility may also have unexplained reasons and this is termed as subfertility or idiopathic infertility [3]. At the present time, there are multiple assisted reproductive technics (ART) available, which provide effective treatment of male subfertility and result in successful fertilization. Nevertheless, genetic and epigenetic defects, as well as abnormalities in sperm chromatin structure, represent a major problem for reproductive medicine, because in contrast to sperm count and motility impairments, sperm chromatin and DNA failures cannot be overcome, even, by applying intracytoplasmic sperm injection (ICSI).

During spermiogenesis, DNA-binding histones are replaced by protamines. As nucleoprotamine package DNA is approximately ten times more efficient than nucleohistones, transcription stops in elongating spermatids. In men, histone to protamine exchange is incomplete and the remaining histones are located in the annular region and are highly acetylated [4,5]. Although histone acetylation is a characteristic feature of transcriptional active genes, it is known that spermatozoa are transcriptionally inactive [6]. This obvious inconsistency resulted in the hypothesis that histone acetylation represents an epigenetic mark that is transmitted from sperm to oocyte and involved in the regulation of gene expression in the early embryo. In the murine zygote, histone variants H2AX, H3.3, H4K8ac, and histone H4 acetylated at lysine 12 (H4K12ac) were demonstrated prior to full decondensation of the sperm nucleus and any substitutions by maternal factors [7] indicated that they must have originated from the sperm [7-9].

Hammoud *et al*. [10] and Arpanahi *et al*. [11] were the first who performed deep sequencing and comparative genome hybridization of the sperm genome, respectively, and reported that remaining nucleosomes are enriched at loci of developmental importance, including imprinted gene clusters, HOX gene clusters, and microRNA clusters and promoters of developmental transcription and signaling factors. Developmental loci, in addition, were associated with H3K4me2 and H3K4me3 [10]. Authors postulated that the transmission of paternal epigenetic information by modified histones may be dependent on the binding position around the transcriptional start site (TSS) and the size of the region interacting with a distinct modification. This hypothesis was supported by ontology analyses showing that developmental gene functions are more over-represented among genes which are more broadly marked by H3K27me3 in spermatozoa. Subsequently, our group demonstrated that binding intensities for H4K12ac, on average, were highest between 0 and 2 kb downstream to the TSS [12]. Using data from Hammoud *et al*. [10], Vavouri and Lehner [13] reported that nucleosome retention occurs preferentially at GC-rich sequences. This was confirmed by Hisano *et al*. [14] in mice. A recent study in men and bulls demonstrated that the vast majority of retained nucleosomes are located within distal intergenic regions and introns are associated with centromere repeats and retrotransposons, while 5′-UTRs, 3′-UTRs, TSSs, and TTSs were depleted from nucleosomes [15]. At the same time, Carone *et al*. [16] reported different results for nucleosome retention in mice depending on the method of nucleosome collection.

Besides modification of histones, methylation of sperm DNA may be involved in the chromatin repackaging process. Three structurally distinct chromatin configurations may occur as follows: histone-packaged hypomethylated DNA, histone-packaged methylated DNA, and protamine-packaged hypomethylated DNA [17]. Bioinformatic alignments of H4K12ac chromatin immunoprecipitation assay in combination with microarray (ChIP-chip) data [11] with sperm methylome [18] provided the first overview of H4K12ac association with methylated or unmethylated gene promoters. On a genome-wide scale, H4K12ac-associated sequences closely co-localized with promoter hypermethylation clusters. As global methylation of gene sequences is a known feature of mature sperm chromatin, the correspondence between gene density and DNA methylation was not unexpected. However, exceptions to the trend for promoter methylation include the developmentally important HOX gene clusters and the PAX genes [17].

Based on our previous studies [7,13], where we analyzed genome-wide binding sites for H4K12ac in the sperm of healthy donors, we now investigated whether subfertile patients with incomplete sperm chromatin condensation exhibit aberrant binding of H4K12ac to selected gene promoters. To analyze whether epigenetic marks may be transmitted from the sperm to the zygote, we investigated the co-localization of H4K12ac with 5-methylcytosine (global methylation) and 5-hydroxymethylcytosine in murine sperm and early embryos. To evaluate whether DNA methylation of developmental gene promoters associated with H4K12ac may be involved in male infertility, we compared methylation levels of single CpGs within CpGs islands of nine selected H4K12ac-bound promoters (AFF4, AXIN1, EP300, LRP5, RUVBL1, USP9X, NCOA6, NSD1, POU2F1) between healthy donors and subfertile patients.

## Results

### Loss of H4K12ac promoter binding in sperm DNA of subfertile patients

Based on the results obtained from ChIP-chip analysis with H4K12ac from fertile donors and semen samples of subfertile patients, as well as gene ontology classifications, we were able to select fertility/developmentally relevant sperm promoters that lack interaction with H4K12 in sperm DNA of subfertile patients. Selected gene promoters are listed in Table 1.

**Table 1:** **Summary of H4K12ac lacking promoters in spermatozoa of subfertile patients**

**Function of promoters depleted for H4K12ac binding in sperm chromatin of subfertile patients**		**References**
AFF4	AF4/FMR2 family member 4, AFF4-deficient mice male mice are sterile with azoospermia and their outcomes died in utero and neonatally with impaired embryonic development.	Urano *et al*. [39]
NCOA6	Nuclear receptor co-activator 6; involved in the co-activation of different nuclear receptors, such as for steroids (GR and ERs), retinoids (RARs and RXRs), thyroid hormone (TRs), vitamin D3 (VDR), and prostanoids (PPARs).	Li and Xu [40]
RUVBL1	RuvB-like 1, TATA box-binding protein-interacting protein, component of the NuA4 histone acetyltransferase complex.	Yamauchi *et al*. [41], Choudhary *et al*. [42]
NSD1	Histone methyltransferase, methylates ‘Lys-36’ of histone H3 and ‘Lys-20’ of histone H4 (*in vitro*). Transcriptional intermediary factor capable of both negatively or positively influencing transcription. Defects in NSD1 are a cause of Beckwith-Wiedemann syndrome.	Tatton-Brown and Weksberg [43], Crea [44], Baujat *et al*. [45]
EP300	Histone acetyltransferase p300; regulates transcription via chromatin remodeling.	Zhang *et al*. [46]
LRP5	Low-density lipoprotein receptor-related protein 5; forms phosphorylated oligomer aggregates on Wnt-signaling. Required for posterior patterning of the epiblast during gastrulation.	Joiner *et al*. [47]
POU2F1	POU domain, class 2, transcription factor 1. Transcription factor that binds to the octamer motif (5′-ATTTGCAT-3′) and activates the promoters of the genes for some small nuclear RNAs.	Latchman [48]

**Figure 1:**
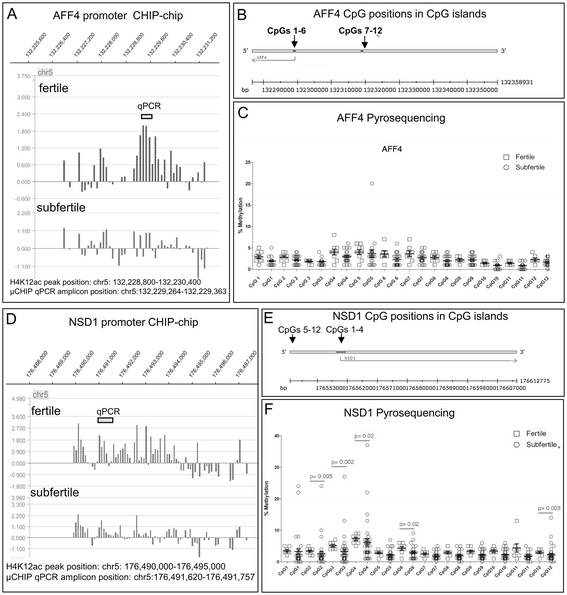
**Methylation analysis of the selected H4K12ac interacting promoters. (A,** **D)** The enrichment of binding sites for H4K12ac AFF4 and NSD 1 in sperm chromatin of fertile and subfertile men (ChIP-chip assay Hg18 NimblGene). ChIP-chip, chromatin immunoprecipitation assay in combination with microarray. Each feature on the array had a corresponding scaled log2 ratio that was calculated from the input signal Cy3 for the total of chromatin and IP probe - Cy5, which were co-hybridized to the array. The log2 ratio was computed and scaled to the center the ratio data around zero. Scaling was performed by subtracting the bi-weight mean of the log2 ratio values for all features on the array from each log2 ratio value. The binding sites for H4K12ac to sperm chromatin were detected by searching for four or more oligo probes whose signals were above the specific cut off values, ranging from 90% to 15%, using a 500-bp sliding window. The ratio data were randomized 20 times, and each peak was assigned a false discovery rate (FDR) score based on the randomization. The lower the FDR score, the more likely the peak corresponded to a H4K12ac binding site. Data are visualized using SignalMap browser (NimbleGen). **(B, E)** Genomic position of analyzed CpGs within CpG islands of AFF4 and NSD1 - pyrosequencing. **(C, F)** Methylation levels of each investigated CpG in sperm DNA of fertile and subfertile patients.

Our genome-wide analysis showed no significant peak corresponding to an enrichment of the binding site (FDR-false discovery rate) of all of selected promoters AFF4 and NSD1 (Figure 1A, D). Peaks of other investigated promoters POU1F2, NCOA6, LRP5, EP300, USPX9, and RUVBL1 are shown in Additional file 1.

**Figure 2:**
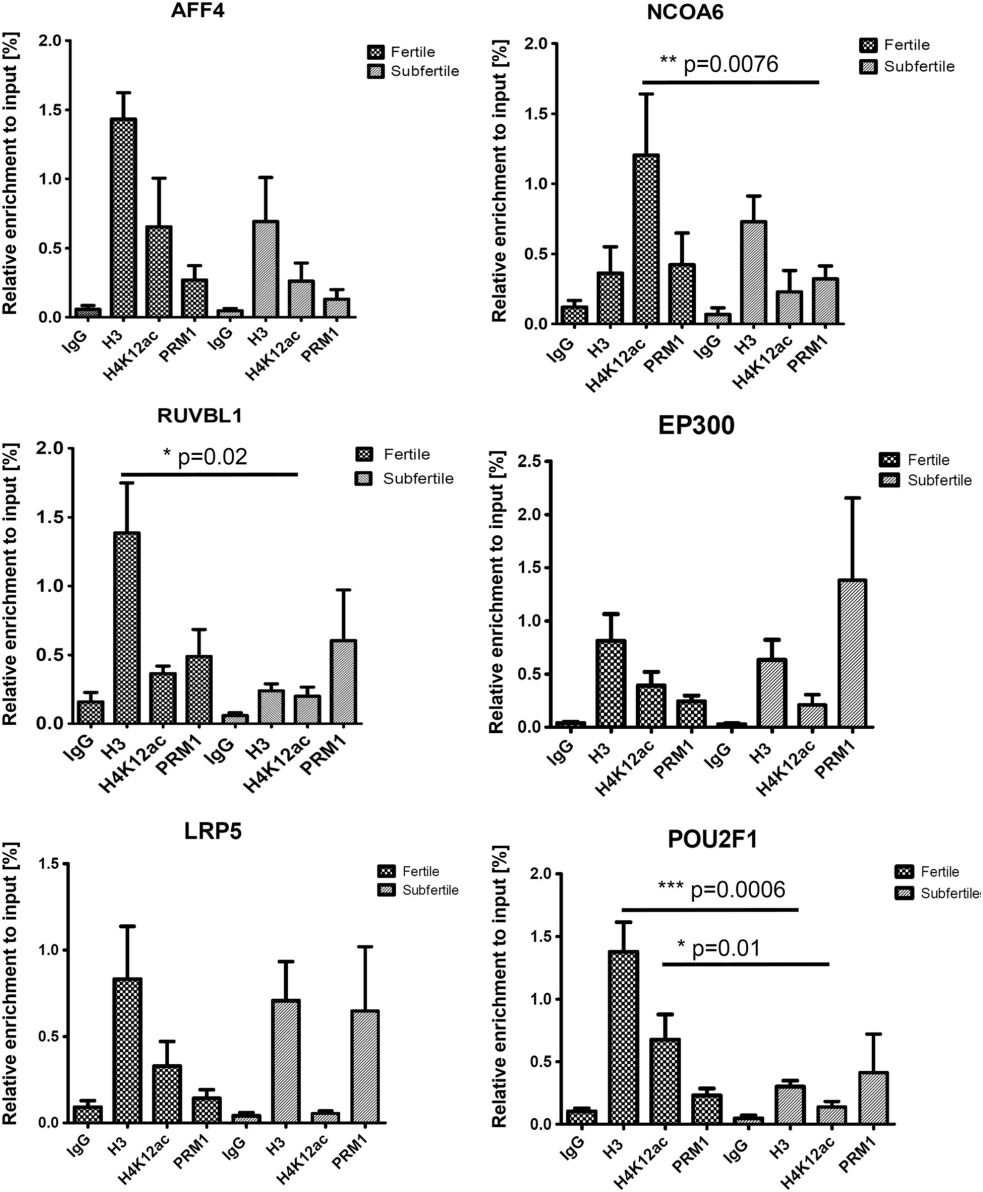
**Validation of enriched promoters with μChIP in combination with real-time polymerase chain reaction (PCR) between sperm of fertile donors and subfertile patients.** Results from ChIP assay with anti-H4K12ac antibodies, with anti-protamine-1 in immunoprecipitated DNA of fertile donors (*n* = 5) and subfertile patients (*n* = 8). Unmodified H3 was used as a positive and IgG as a negative isotype control. Loss of interaction for H4K12ac was detected in selected promoters; however, a significant difference was observed in NCOA6, RUVBL1, and POU2F1. Depletion of H4K12ac binding is potentially replaced by either unmodified histone H3 or protamine 1.

**Figure 3:**
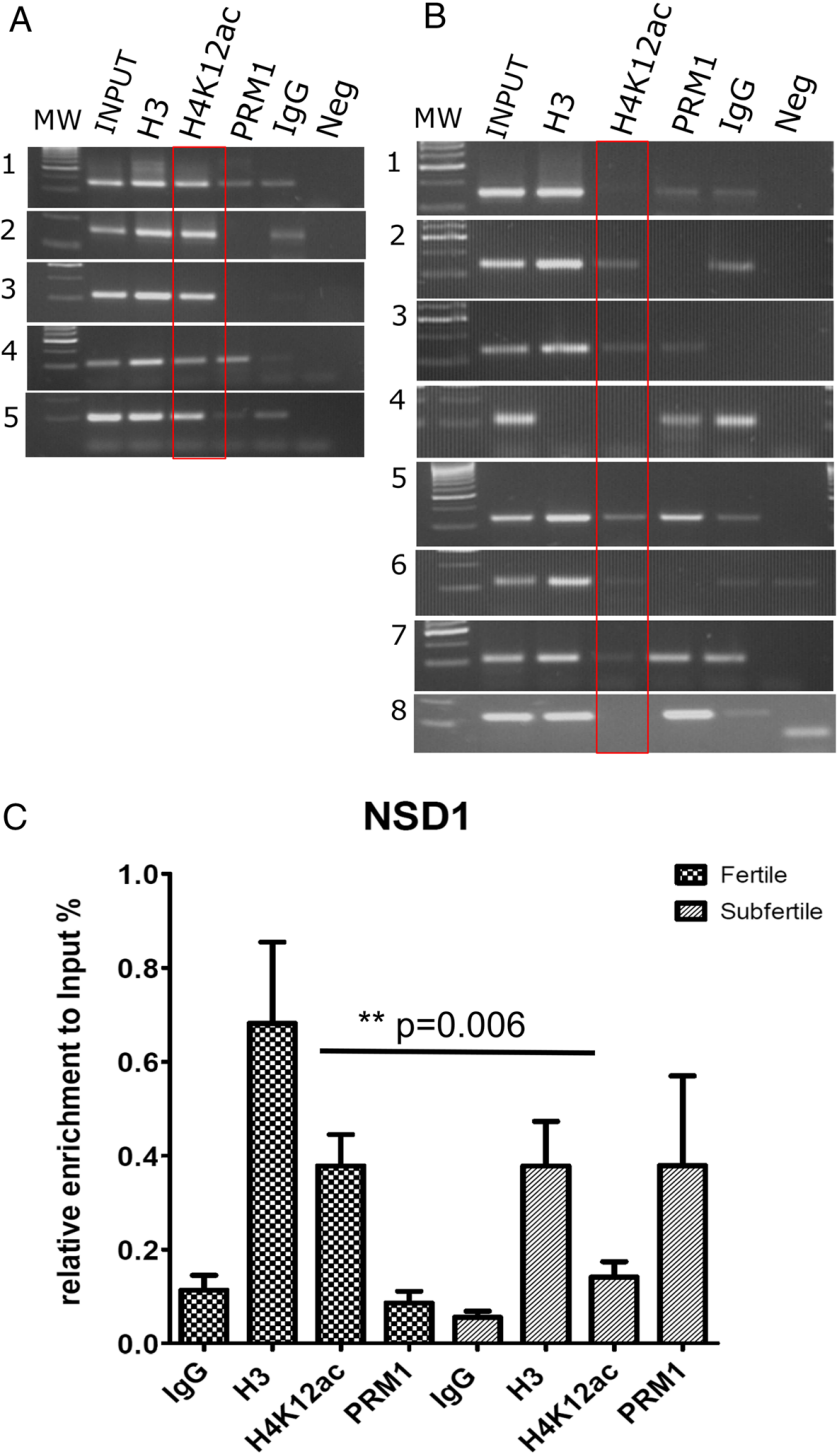
**μChIP assay in combination with real time qPCR demonstrating loss of association of NSD1 promoter sequence with H4K12ac in subfertile patients with aberrant sperm chromatin condensation. (A)** Representative qPCR demonstrating enrichment of μChIP experiments with anti-H4K12ac antibodies, with anti-protamine-1 antibodies from input material in individual fertile donors (*fc* = 5) and **(B)** subfertile patients (*n* = 8). Unmodified H3 was used as a positive and IgG as a negative, isotype control. **(C)** Enrichment of the immunoprecipitated sample from fertile and subfertile men compared to input materials was calculated as follows: ΔCt = Ct (input) - Ct (immunoprecipitated sample) and % total = 2^ΔCt^ × 10 (according to 10% input chromatin of total immunoprecipitated chromatin). Depletion of binding and an increase of protamine occupancy to the NSD1 promoter were detected.

Using μChIP, which is optimized for minimum input material corresponding to 1 million sperm cells, we identified binding sites for nine H4K12ac-associated or depleted promoters in sperm of subfertile patients and fertile donors, respectively. μChIP assay showed an overall decrease in the binding capacity of H4K12ac to the investigated DNA sequences in subfertile patients when compared to fertile donors (Figure 2). A significant decrease of enrichment has been detected in NCOA61 (*P* = 0.0076), POU2F1 (*P* = 0.01), and NSD1 (*P* = 0.006). In AFF4, RUVBL1, and POU2F promoters, the decrease of H4K12ac enrichment was accompanied by a decrease of H3 enrichment (Figure 2). Regarding the NSD1 promoter, we observed enrichment of H4K12ac in all ejaculates from healthy donors (*n* = 5) (Figure 3). In contrast, in four out of eight subfertile patients, no enrichment of the NSD1 promoter could be observed (Figure 3B). Reduced enrichment was observed in eight patients. Interestingly, the enrichment of unmodified histone H3 was not altered in the subfertile group. In four out of eight patients, a lack of H4K12ac binding was possibly compensated by protamine enrichment to the NSD1 locus (Figure 3C).

### H4K12ac-associated promoters are poised by DNA hypomethylation

**Figure 4:**
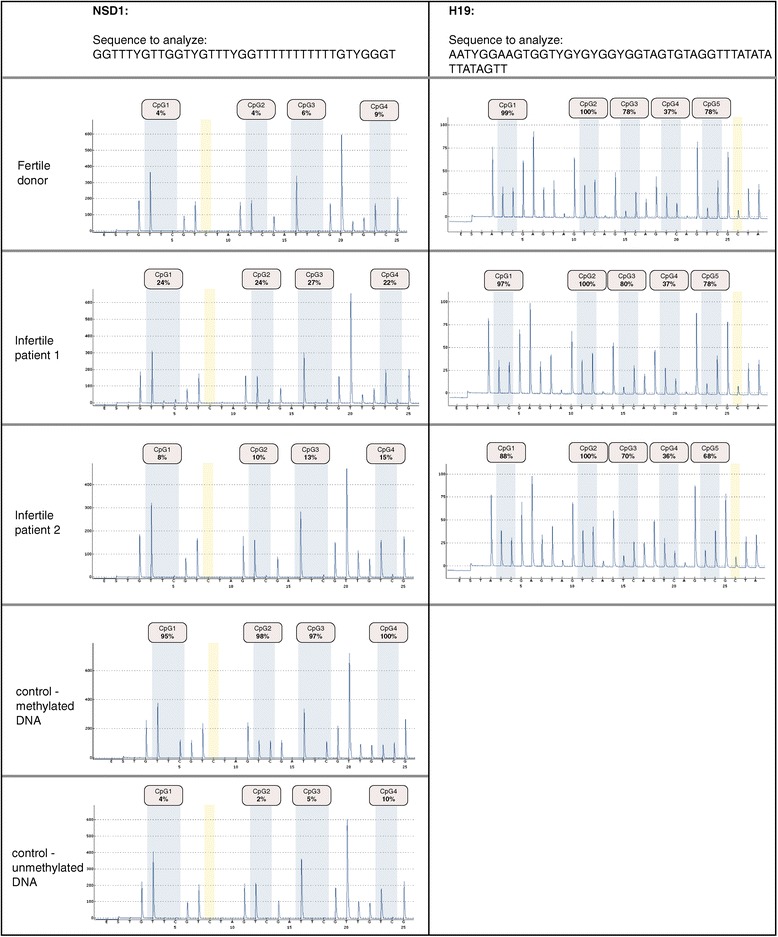
**Pyrograms showing methylation level for each cytosine within the investigated sequences in spermatozoa of one fertile donor and two selected infertile patients.** In contrast to the hypomethylated promoter region of NSD1, paternally imprinted and hypermethylated H19 locus is presented as a control. Pyrosequencing using fully methylated and fully unmethylated DNA was carried out for the internal control.

Investigating the level of a single CpG methylation by using a highly sensitive pyrosequencing method, we could demonstrate the hypomethylation of DNA in all H4K12ac interacting promoters. For each promoter, the region containing 6 to 12 CpGs was covered by pyrosequencing of 1 to 3 polymerase chain reaction (PCR) products. The number of analyzed CpGs was as follows: AFF4 - 12 CpGs; EP300 - 5 CpGs; LRP - 6CpGs; NCOA6 - 6 CpGs; RuVBL1 -5 CpGs; USP9X - 5CpGs; POU2F1 - 5CpGs; and NSD1 - 12 CpGs. For an internal control, a fully methylated and unmethylated sodium bisulphite converted DNA was included. In addition, pyrosequencing of paternally imprinted and hypermethylated locus of H19 was carried out (Figure 4). A low methylation level of each CpGs (range 5% to 25%) was detected in the sperm of donors and subfertile patients. We observed a significant decline of methylation in the AFF4 promoter in position CpG 1 (2.8% ± 1.2% vs. 1.9% ± 0.9%; *P* = 0.016) in the subfertile group (Figure 1). In the EP300 promoter, consistent 5% methylation was detected in the group of fertile men, while in several subfertile men, methylation increased up to 17%. A significant decrease in methylation was observed in CpG 3 (6.44% ± 2.7% vs. 4.6% ± 2.5%; *P* = 0.01) and CpG4 (4.0% ± 1.5% vs. 3.0% ± 1.7%; *P* = 0.04) of the LRP promoter (Additional file 1). The value of CpGs methylation in NCOA6, RuVBL1, USP9X, and POU2F1 promoters did not exceed 5% on average, and the standard deviations were low in the group of fertile donors. In the NSD1 promoter, there were significant differences in the position of CpG2 (3.3% ± 1.0% vs. 2.54% ± 4.1%; *P* = 0.005) and CpG 11 (4.3% ± 3.7% vs. 1.7% ± 1.0%; *P* = 0.003). We observed a different methylation pattern among subfertile patients; however, the CpG methylation levels varied between 5% to 20%, which is considered as hypomethylation (Additional file 1).

**Figure 5:**
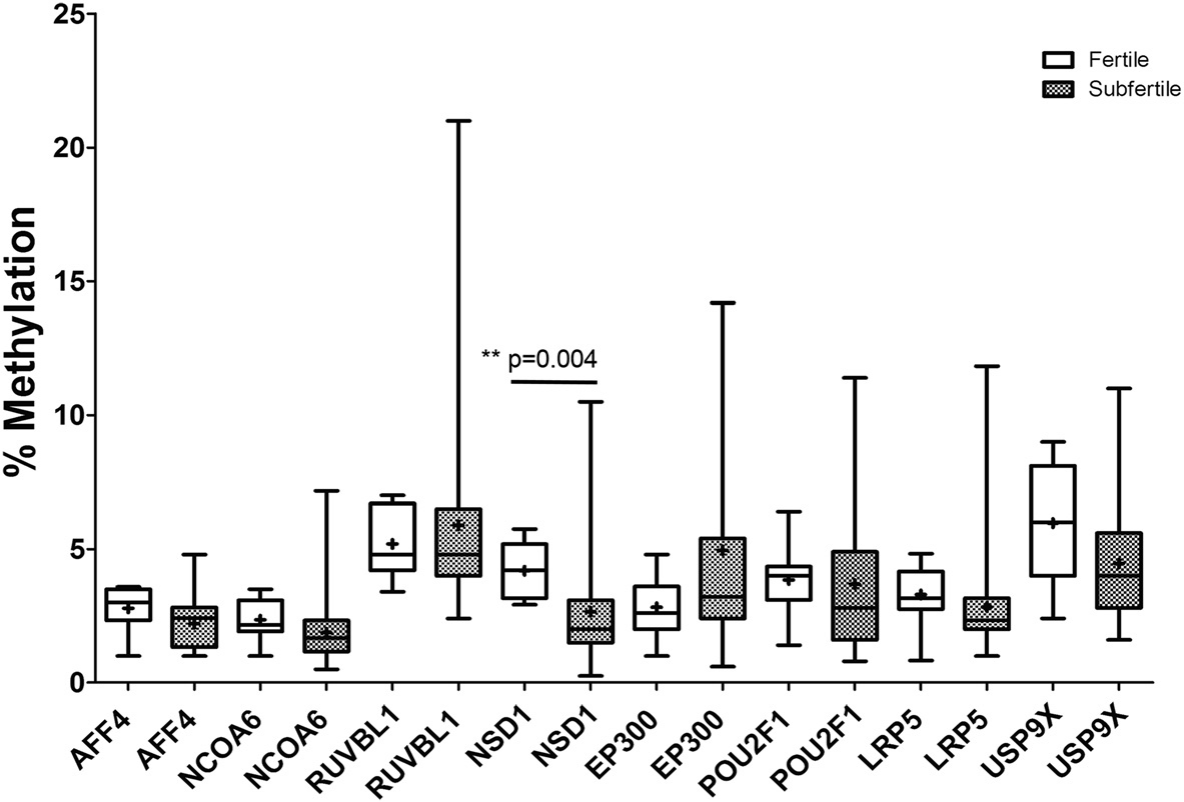
**DNA methylation level of investigated H4K12ac interacting promoters in spermatozoa from** ***n*** **= 8 fertile donors and** ***n*** **= 39 subfertile patients - pyrosequencing.** While the group of fertile donors represented a stable methylation pattern, a large disproportion and high standard deviation were observed in the group of subfertile men.

**Figure 6:**
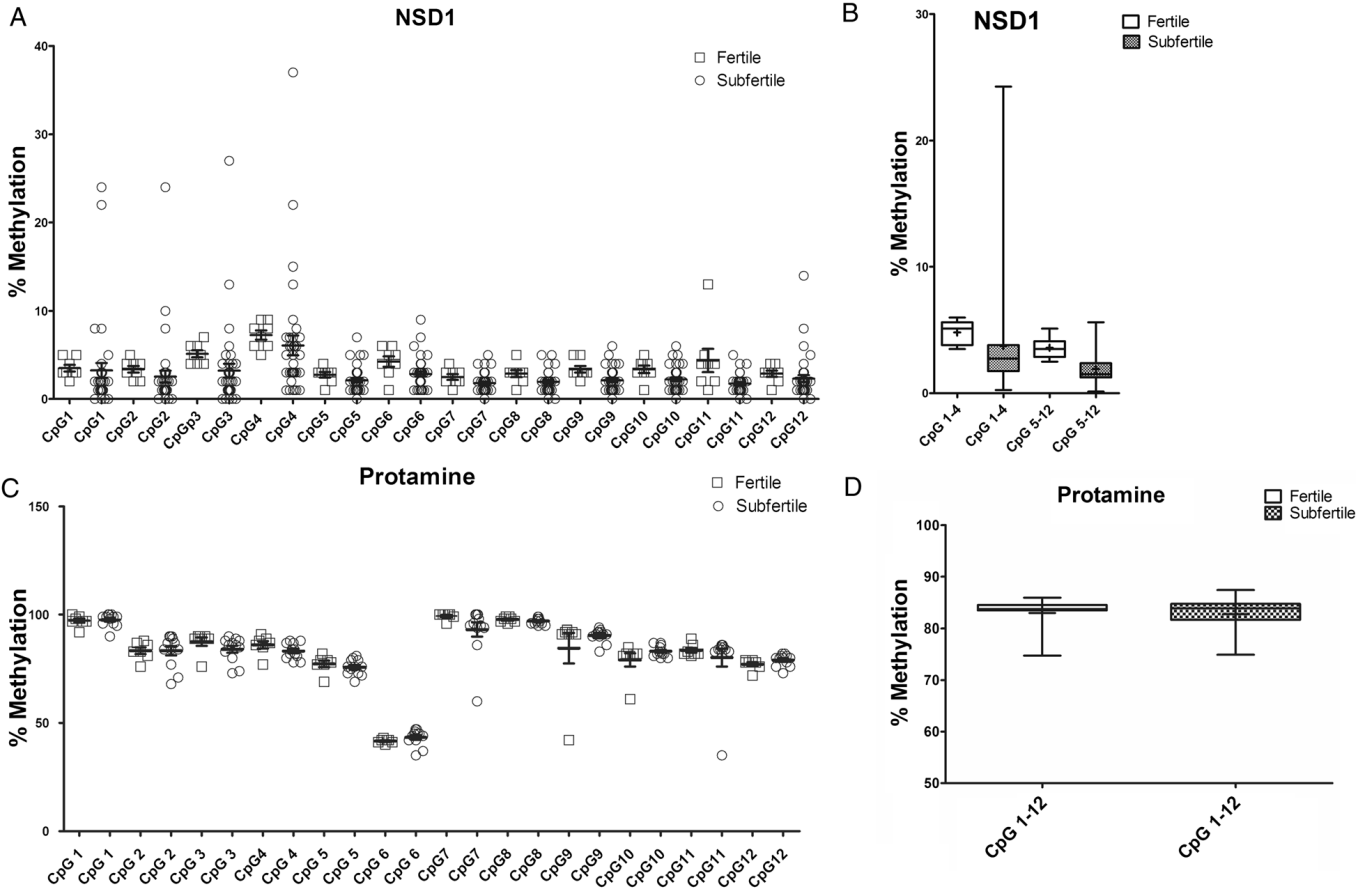
**The methylation level in each cytosine in sperm DNA was analyzed by pyrosequencing. (A)** DNA methylation in 12 cytosines (CpG) in H4K12ac-associated NSD1 promoter in fertile donors and subfertile patients. **(B)** Averaged DNA methylation in 1 to 4 CpGs and 5 to 12 CpGs of NSD1 promoter in fertile and subfertile men. **(C)** DNA hypermethylation in 12 CpGs in sperm-specific protamine promoter. **(D)** Averaged methylation from 12 CpGs in protamine promoters showing no significant difference between fertile and subfertile sperm.

In all H4K12ac-associated promoters, no significant difference was detected regarding the cumulative CpG methylation of the investigated promoters despite NSD1 (4.1% ± 1.0% vs. 2.6% ± 1.9%; *P* = 0.004) (Figure 5). The methylation of the protamine promoter, that is not associated with H4K12ac, displayed an opposite methylation pattern. Twelve CpGs of protamine 1/2 common CpG island have been highly methylated (40% to 100%); however, no changes in subfertile patients were detected (Figure 6).

The association of DNA methylation of H4K12ac-binding promoters with clinical parameters (Table 2) did not reveal any significant correlations despite the POU2F promoter (Table 3). POU2F DNA methylation correlated negatively with progressive sperm motility (*R* = −0.43; *P* = 0.023). Although not significant, but still noticeable, negative correlation could be observed in POU2F DNA methylation and in sperm concentration (*R* = −0.30; *P* = 0.121). A similar trend of negative correlation could be seen with POU2F DNA methylation and the fertility rate (*R* = −0.33; *P* = 0.08). Protamine DNA methylation as a ‘non-histone’ associated promoter was strongly correlated with the fertility rate (*R* = 0.71; *P* = 0.003), and a tentative positive correlation could be observed with protamine DNA methylation and sperm concentration (*R* = 0.41; *P* = 0.138).

**Table 2:** **Semen characteristics of 39 men with unexplained infertility**

	**Mean**	**SD**	**Median**	**Minimum**	**Maximum**
Sperm parameters of subfertile patients and ICSI outcomes					
Volume (ml)	4.38	2.46	4	0.5	10
Sperm count (× 10^6^/ml)	41.12	29.9	32	3.9	140
Total sperm count (× 10^6^)	150.2	120	140.7	21.45	702
Total motility (%)	36.7	15.94	35	67	10
Progressive motility	7.12	5.7	7	0	18
Morphology normal forms (%)	10.03	5.16	10	24	2
Aniline blue staining (2 + 3)	48.4	15.8	45	20	76
ICSI outcomes of subfertile men					
Number of oocytes	6.94	3.96	7	22	2
First pronucleus stage	4.20	4.0	2.51	13	1
Second pronucleus stage	3.79	2.17	3.0	10	1
Fertility rate (%)	58.44	24.16	57.14	20	100
Number of embryos transferred	2.17	0.60	2.0	3	1

**Table 3:** **Correlations between methylation in sperm DNA within different H4K12ac-associated promoters, ejaculate parameters, and fertility rates**

**Promoter**	**Sperm concentration (** **×** **10** ^**6**^ **/ml)**	**Sperm motility**	**Sperm morphology**	**Fertility rate (ICSI)**
AFF4	*R* = −0.25; *P* = 0.232	*R* = −0.04; *P* = 0.821	*R* = 0.08; *P* = 0.692	*R* = −0.272; *P* = 0.18
NCOA6	*R* = −0.07; *P* = 0.677	*R* = 0.24; *P* = 0.183	*R* = 0.15; *P* = 0.387	*R* = 0.06; *P* = 0.72
RUVBL1	*R* = −0.05; *P* = 0.767	*R* = −0.03; *P* = 0.870	*R* = −0.28; *P* = 0.138	*R* = 0.36; *P* = 0.05
NSD1	*R* = −0.32; *P* = 0.071	*R* = −0.05; *P* = 0.976	*R* = −0.17; *P* = 0.326	*R* = 0.22; *P* = 0.21
USP9X	*R* = −0.03; *P* = 0.865	*R* = 0.01; *P* = 0.943	*R* = −0.39; *P* = 0.072	*R* = 0.17; *P* = 0.42
POU2F	*R* = −0.30; *P* = 0.121	*R* = −0.43; *P* = 0.023*	*R* = −0.24; *P* = 0.217	*R* = −0.33; *P* = 0.08
Protamine	*R* = 0.41; *P* = 0.138	*R* = 0.12; *P* = 0.689	*R* = −0.12; *P* = 0.976	*R* = 0.71; *P* = 0.003**

Significant values when *P ≤ 0.05 and **P ≤ 0.01.

### Paternally derived H4K12ac pattern resembles global DNA demethylation in male pronucleus shortly after fertilization (mouse model)

**Figure 7:**
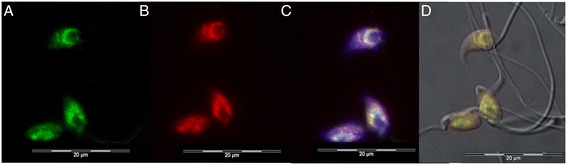
**Immunofluorescent labeling of H4K12ac and 5-methylcytosine (5mC) in mouse sperm nucleus.** Double stained spermatozoa with anti-H4K12ac antibody (green) **(A)**, anti-5mC antibody (red) **(B)**, merged with DAPI **(C)**, and merged with DIC **(D)**.

In order to study the transmission of epigenetic marks from paternal chromatin (predominantly H4K12ac and DNA methylation) to the embryo, we used a mouse *in vitro* fertilization model followed by indirect immunofluorescence. Spermatozoa before fertilization, and pronuclei in early developmental pronuclear stages (PN3-PN5) prior to fusion and division, were subjected to immunostaining. In sperm, the fluorescence signal was clearly detectable in the postacrosomal region of the sperm head over the central part of the nucleus (green) (Figure 7). Interestingly, we identified the same localization for 5-methylcytosine (5mC) signal (red) (Figure 7), suggesting that these epigenetic marks occupy the same compartment of the mouse sperm nucleus.

**Figure 8:**
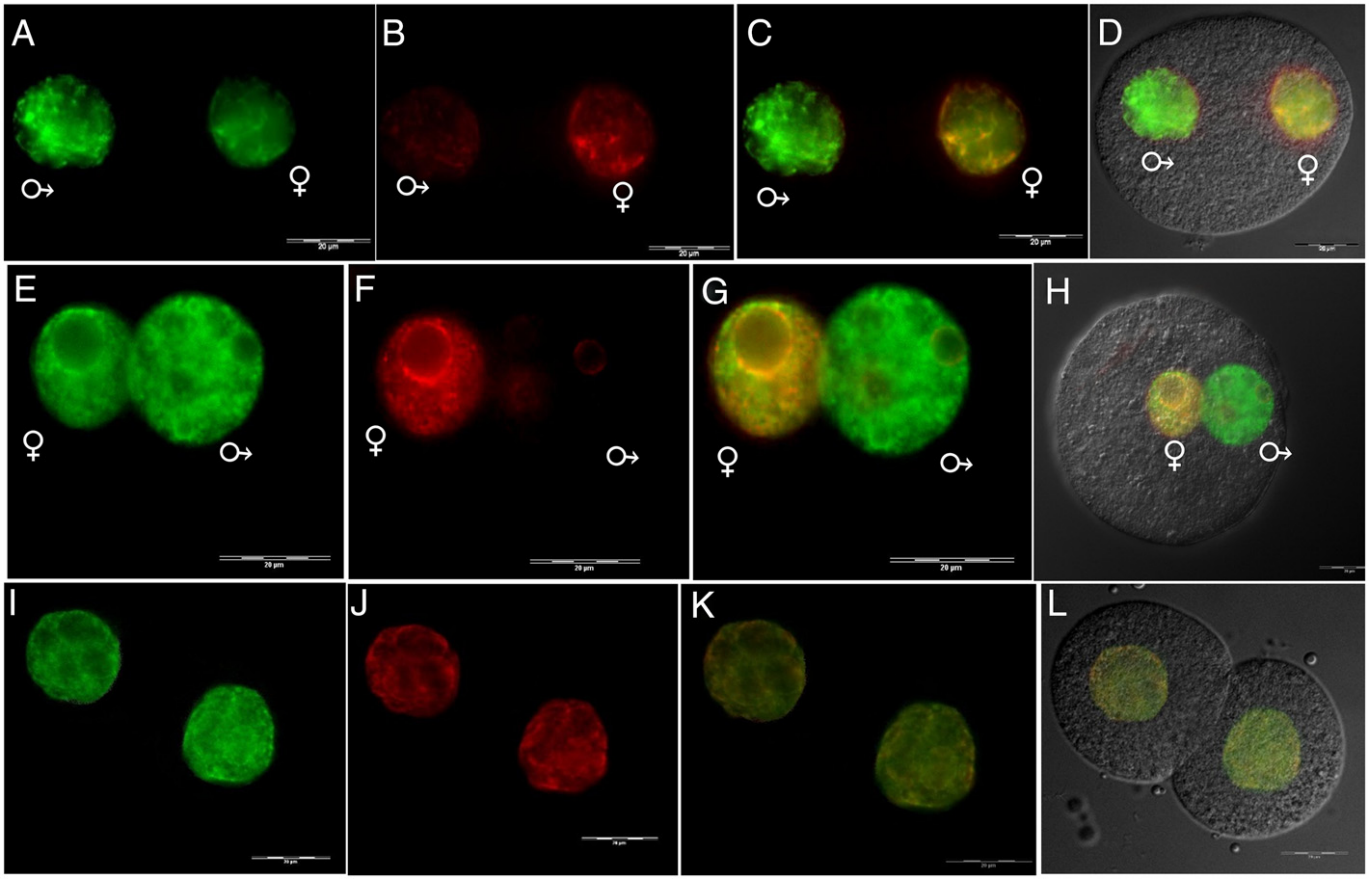
**Immunofluorescent labeling of H4K12ac and 5-methylcytosine (5mC) in mouse early embryos.** Double stained embryos with anti-H4K12ac antibody (green) **(A, E, I)**, anti-5mC antibody (red) **(B, F, J)**, merged **(C, J, K)**, merged with DIC **(D, H, L)**. **(A-D)** Pronucleus at stage PN3. **(E-H)** Pronucleus at stage PN5. **(I-L)** Two-cell embryo. Maternal pronucleus (♀) and paternal pronucleus (♂). Scale bar represents 20 μm.

**Figure 9:**
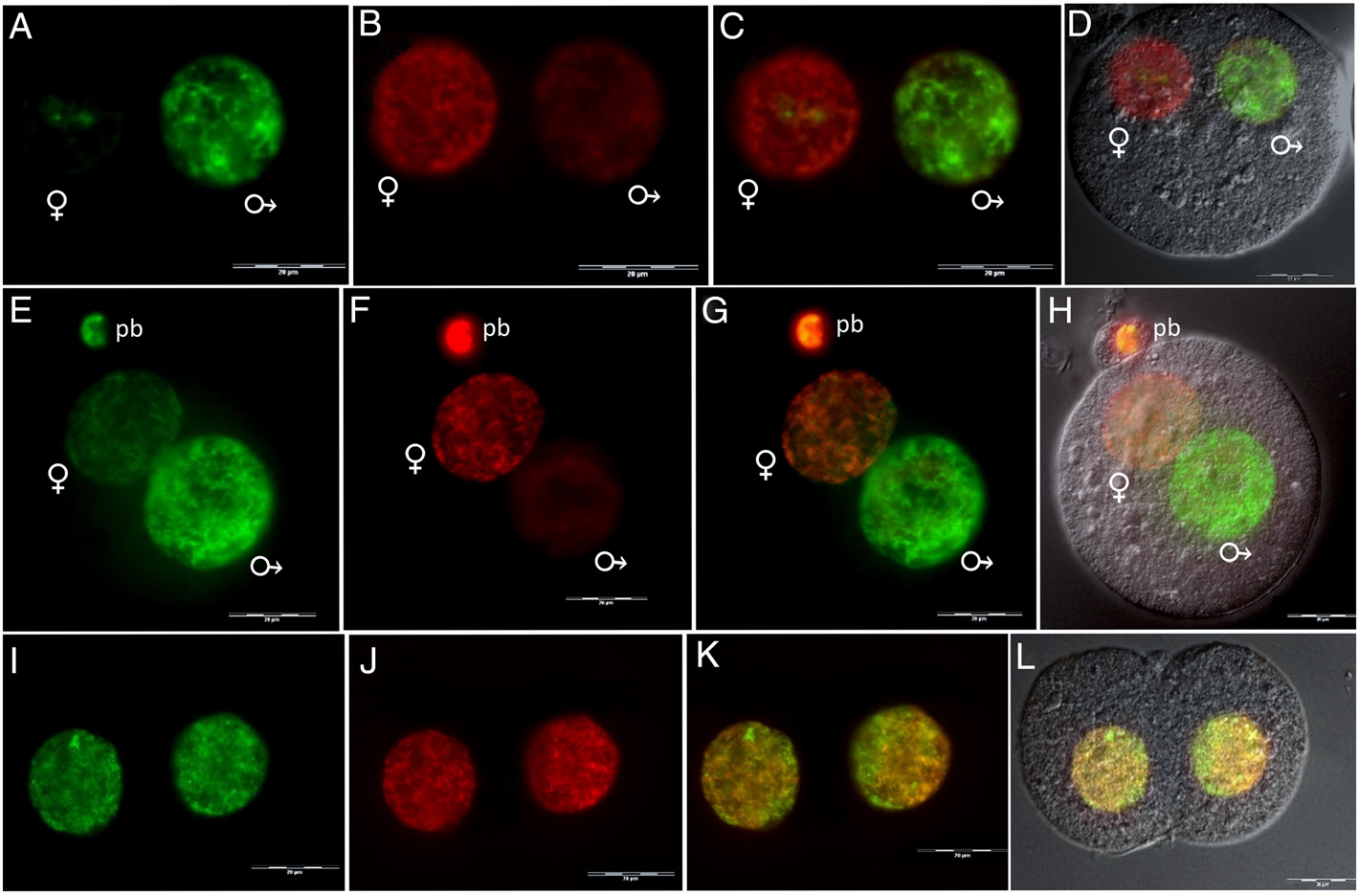
**Immunofluorescent labeling of 5-hydroxymethylcytosine (5hmC) and 5-methylcytosine (5mC).** Double stained embryos with anti-5hmC antibody (green) **(A, E, I)**, anti-5mC antibody (red) **(B, F, J)**, merged **(C, J, K)**, merged with DIC **(D, H, L). (A-D)** Pronucleus at stage PN3. **(E-H)** Pronucleus at stage PN5. **(I-L)** Two-cell embryo. Maternal pronucleus (♀) and paternal pronucleus (♂). pb, polar body. Scale bar represents 20 μm.

The localization of H4K12ac in mouse-fertilized eggs with clearly established pronuclei was analyzed. The male and female pronuclei were distinguished by their naturally differing size, with the male pronucleus being larger than the female pronucleus. Starting from the time when pronuclei are formed, the paternal one exhibits a strong signal for H4K12ac (Figure 8A), while in the maternal pronucleus, there is a continual increase of H4K12ac until pronuclei fusion (Figure 8A, E, I). Simultaneously, there is a continual decrease of the DNA methylation state in the paternal pronucleus indicated by an increase of the 5-hydroxymethylcytosine (5hmC) signal (Figure 9A, E) and a decrease of the 5mC signal (Figure 9B, F). Meanwhile, the maternal pronucleus becomes widely methylated (Figure 9B, F). DNA demethylation and acetylation on lysine K12 of histone H4 are genome activating modifications underlying differences in the transcription activity of the pronuclei. After gamete fusion in the two-cell stage, a homogenous staining for H4K12ac and 5mC was observed (Figure 8I, J, K). A similar pattern was detected for 5mC and 5hmC (Figure 9I, J, K). Pronuclei of parthenogenetically activated oocytes show the ability to substitute paternal H4K12ac, and the degree of DNA demethylation is higher than in the maternal pronucleus of the control zygote (Additional file 2). This fact suggests the important role of H4K12ac for the accumulation of transcription factors and the regulation of gene expression during early embryogenesis.

## Discussion

The sperm epigenome has recently been the subject of intensive investigations in research laboratories of reproductive medicine/biology. Although extensive progress has been made in the understanding of sperm chromatin packaging into protamine toroids [17], genome-wide histone retentions [10,11,19] and DNA methylation [20] epigenetic factors of infertility and their effect on embryogenesis still remain to be elucidated. This study demonstrates that a loss of binding sites for H4K12ac in selected developmentally important promoters in the sperm of subfertile men gives rise to the idea of a possible epigenetic aberration that sperm of subfertile men transport to the oocyte. Pyrosequencing analysis revealed hypomethylation of all H4K12ac interacting promoters. This methylation pattern has been conserved in the population of subfertile sperm. While sperm DNA methylation of normozoospermic men displayed low standard deviation, a higher variability in the percentage of methylation was detected in the promoters of subfertile men; however, 30% of methylation was not exceeded. In contrast, cytosines within the CpG island of the protamine gene were hypermethylated, and no changes between healthy donors and subfertile patients were detected. Furthermore, the relevance of sperm derived H4K12ac for pronucleus formation and its resemblance to DNA demethylation was confirmed by immunofluorescence in the mouse model.

The loss of H4K12ac enrichment in a genome-wide manner and in specific loci may have its origins in either improper remodeling during spermatogenesis or a decreased activity of histone acetyl transferases (HATs) and histone deacetylases (HDACs).

Interestingly, a reduced binding, of H4K12ac to EP300 and LRP5 promoters, was compensated by protamines. On the other hand, sperm phenotypes with over 50% aniline blue staining suggest that there is an increased amount of total histones in subfertile spermatozoa. The principle of the aniline blue staining is to differentiate between lysine-rich histones and arginine/cysteine-rich protamines. Aniline blue stain lysines of histone-rich nuclei of immature spermatozoa, where protamine-rich nuclei of mature spermatozoa containing relatively low levels of lysine, do not display staining [21]. The reduced binding of H4K12ac to developmentally important promoters might be explained by a possible loss of lysine 12 acetylation. The acetyl group counteracts the positive charge of the epsilon-amino group of the lysine, reducing the overall charge of histones, and decreases their affinity for the negatively charged DNA. This causes the opening of chromatin and the facilitation of the recruitment of the transcriptional machinery [22]. As sperms are transcriptionally inactive cells, interaction with H4K12ac within developmentally important promoters may facilitate the recruitment of factors initiating transcription.

Whether acetylation itself is only associated with gene activation is still not certain because it has been recently shown that protamines also contain acetyl groups [23]. The data also identify post-translational modifications, such as S42 and K49 acetylation of protamine-1 and K64 acetylation on protamine-2 [23]. The fact that protamines carry marks associated with transcriptional activation is intriguing, as these proteins are thought to ensure tight packaging of the DNA in sperm cells and contribute to transcriptional silencing [24].

The clinical aspects of histone modifications interacting with promoters in sperm of infertile men are not yet well addressed in the literature. Besides our group, Hammoud *et al*. (2011, 2009) [10,25] have evaluated the differences in sperm histone modifications between healthy donors and men who displayed aberrant protamine P1/P2 ratio and who had an unexplained poor embryogenesis during *in vitro* fertilization (IVF) [26]. Analyzing H3 lysine 4 methylation (H3K4me) or H3 lysine 27 methylation (H3K27me), the authors reported a highly similar localization pattern in the gametes of infertile men compared to fertile men. However, there was a reduction in the amount of H3K4me or H3K27me retained as developmental transcription factors and certain imprinted genes, which appear to be in agreement with our results. Analyzing the distribution of H3K9ac in the sperm genome, using ChIP in combination with ENCODE array, we identified interactions in developmentally relevant promoters (FAM50A, CAV1, HOXA13, TH, MET, SF1, AFF4, HCFC1, INHA, ELL3, WNT2, CTTNBP2, HOXA10, THOC2, AXIN1) and exonic and intergenic sequences. In our previous study, random distribution and depletion of H3K9ac interaction in many loci (for example, CTSD, FLNA, MCF2L, PLXNA3, SG3GLB2, TH) of infertile men sperm have been confirmed [27]. Findings obtained from our own and other investigators’ studies suggest an aberrant distribution of histone retentions in the genome of subfertile men, and this seems to be a common observation for several histone modifications.

To examine whether a depletion of binding sites for H4K12ac in developmentally important promoters might result in a change in DNA methylation, pyrosequencing of 9 candidate developmental loci that lacked or had significantly reduced levels of H4K12ac and the protamine promoter were assessed in 38 subfertile men. H4K12ac promoters of fertile and subfertile men were correlated with DNA hypomethylation; however, they were displaying a more concordant methylation pattern compared to subfertile patients. The loss of binding sites obviously does not cause a gain of DNA methylation, or possibly, these promoters are protected by other histone modifications, as for instance, H3K4me. A most intriguing observation obtained from our results was that single CpG methylation was stable in every selected promoter of the fertile group, while methylation of subfertile patients showed considerable variation within the same group. Methylation studies, using pyrosequencing methods focused on different loci MEST and IGF2⁄H19 ICR1, showed the same effect in the group of subfertile patients [28].

Genome-wide shotgun bisulfite sequencing from sperm DNA provides evidence that the majority of promoters in sperm, similarly to embryonic stem cells, escape methylation. In contrast, repeat elements are strongly methylated in both germ and somatic cells; however, retrotransposons from several subfamilies evade methylation more effectively during male germ cell development [20]. In respect to male infertility, several research approaches have pointed out that imprinted regions were more prone to deregulation of methylated pattern than promoter regions [25,26,29] Nevertheless, the specific promoters including CREM have been shown to possess a significantly higher rate of methylation in patients with abnormal protamination and oligozoospermia compared to the control group [30]. This study investigated a large cohort of 175 oligozoospermic men and demonstrated epimutations in H19-DMR and PEG1/MEST-DMR in 20% and 3% of oligozoospermic men, respectively. The authors identified an amino acid change, in DNMT3A in one case and altered methylation profiles in DNMT3L in eight men, as a possible cause of epimutations. Interestingly, no correlation between the ART outcome and epimutations was found [31].

Finally, in the present study, the relevance of the co-existence of paternally derived chromatin active marks (H4K12ac) and 5hmC in the early pronucleus stages of the mouse embryo was demonstrated. The paternal pronucleus displays a high level of H4K12 acetylation, which interferes with global demethylation. This fact may give rise to the idea that developmentally relevant promoters in sperm occupied with H4K12ac and with a moderate level of methylation may be prepared for the transmission of certain epigenetic marks to the embryo. Recent reports from zebrafish embryos strengthen the hypothesis that sperm DNA methylome, but not oocyte, is inherited during early embryo formation [32]. A previously proposed and generally accepted theory is that the paternal pronucleus is demethylated through the active process by the conversion of 5mC to 5hmC catalyzed by Tet3 and the maternal pronucleus through DNA replication-dependent demethylation [33,34]. According to our results, the paternal pronucleus is widely demethylated before the onset of the first DNA replication at PN3. This happens independently, although, 5hmC in the paternal pronucleus is removed during DNA replication in a passive manner [35]. Indeed, the paternal pronucleus possessed a higher level of 5hmC than the maternal pronucleus, which is evident in all PN stages by strong 5mC labeling. However, the level of 5hmC continually increased during the replication phase. An explanation of this increasing intensity may be that 5hmC could participate in passive demethylation, and the evidence is that Dnmt1 poorly recognizes 5hmC [36].

## Conclusions

Given the well-known heterogeneity of sperm cells within one ejaculate, as well as the multifactorial background of male infertility, we included sperm samples with aberrant sperm chromatin condensation assessed by using aniline blue staining as the subject of the study. Similarly, in order to define the phenotypes of patients with considerable impaired transmission of epigenetic marks to the embryo, we analyzed sperm of patients who had not obtained pregnancy after ICSI treatment. According to our data, H4K12ac is substantially more accessible for epigenetic alteration than cytosine methylation within developmentally relevant promoters. Considering the activating properties of H4K12ac in the regulation of developmentally important genes in the early embryo and the establishment of an open chromatin frame, together with DNA hypomethylation, we speculate that H4K12ac could represent a potential factor for epigenetic-mediated infertility.

## Methods

Ejaculates were obtained from 39 patients, whose female partners took part in an ICSI program in Fertility Centers in Wetzlar and Wiesbaden. All the samples were collected according to the current version of the Declaration of Helsinki, and informed written consent was obtained from every patient before being included in the study. The experimental procedure of using patients’ ejaculates was approved by the Ethics Committee of the Medical Faculty of Justus-Liebig-University (approval 146/06 confirmed on 15 December 2010 for studies of German Research Foundation (DFG), Project 1 of the Clinical Research Unit KFO 181/2).

The control group comprised of nine healthy volunteers with normozoospermia, according to the current World Health Organization (WHO) reference values [37] (WHO, 2010). Hence, semen characteristics from these men are considered as surrogate parameters reflecting normal fertility.

Spermiogram parameters according to the recommendations of WHO (2010) [38] were available (Table 2). Sperm morphology was evaluated according to strict criteria. The total motile sperm count was calculated as the product of volume. The sperm concentration and progressive motility of native semen were assessed. In order to assess the histone and protamine status in sperm, chromatin standard aniline blue staining was conducted [21]. The protamine mRNA ratio was measured from 14 out of 40 patients. Fertility parameters after the ICSI program were registered. The number of oocytes, first and second pronuclear stages, a number of embryos, and the percentage of fertilized oocytes (fertility rate) and pregnancy outcomes were collected (Table 2).

Strict selection criteria have been applied for subfertile patients regarding epigenetic fertility factors, which were investigated in this project. Men diagnosed with idiopathic infertility with aberrant sperm chromatin condensation as assessed by aniline blue staining 2 + 3 > 50%, as well as no pregnancy resulting from ICSI, and 4 out of 20 ICSI cycles ended in an abortion, were included in this study.

### μ-ChIP

For the handling of a small number of sperm cells in subfertile patients, we used a modified version of the Q2ChIP assay. For that, we used a LowCell# ChIP Kit from Diagenode (Cat.No. kch-maglow-A16, Diagenode, Sparta, NJ, USA). Eleven microliters of protein A-coated magnetic beads was washed twice in 22 μl of ice-cold RIPA buffer (10 mM Tris-HCl, pH 7.5, 1 mM EDTA, 1% Triton X-100, 0.1% SDS, 0.1% Na-deoxycholate, 100 mM NaCl). Then, 2.4 μg of the antibodies were added, followed by a 2-h incubation at 4°C. In this study, we used polyclonal IgG ChIP grade (ab46540, Abcam, Cambridge, UK) as a negative control and antibodies against unmodified histone H3 (ab1791, Abcam, Cambridge, UK) as a positive control. ChIP grade rabbit polyclonal antibodies against acetylated histone H4 at lysine 12 (H4K12ac) were used for immunoprecipitation and were purchased from Abcam (ab1761, Abcam, Cambridge, UK). Anti-protamine 1 (PRM1) antibodies (Hub 1 N) were purchased from Briar Patch Biosciences, Livermore, CA, USA.

The respective ejaculate was centrifuged for 10 min at 4,000 rpm/4°C to collect the sperm cells. The supernatant was discarded, and the cells were washed twice with 1 ml PBS+, which means phosphate-buffered saline (PBS) including 20 mM butyrate and protease inhibitor (Complete Mini EDTA-free, 04693150001, Roche, Mannheim, Germany), for 10 min at 4,000 rpm/4°C. Then, 500 μl of PBS+ and 13.5 μl of 36.5% formaldehyde were added for crosslinking. After 8 min at room temperature, 57 μl of 1.25 M glycine was added to stop the fixation. After 5-min incubation followed by centrifugation at 6000 × *g* for 10 min at 4°C, the supernatant was discarded and the cells were washed twice in 0.5 ml of ice-cold PBS+. Then, 120 μl of lysis buffer (50 mM Tris-HCl, pH 8, 10 mM EDTA, 1% SDS, protease inhibitor cocktail, 20 mM butyrate) and glass beads (G1277, Sigma-Aldrich, Seelze, Germany) were added and incubated on ice followed by a treatment of Bioruptor sonicator (UCD-200 TO, Diagenode, Sparta, NJ, USA) (25 cycles of: (30 s ‘ON,’ 30 s ‘OFF’)). After sonication, the samples were supplemented with 480 μl of RIPA ChIP buffer (RIPA buffer including 20 mM butyrate and protease inhibitor) and centrifuged at 12,000 × *g* for 10 min at 4°C. While the supernatant was transferred to a new tube, the pellet was washed with 330 μl RIPA ChIP buffer as before, resulting in a second supernatant that was pooled with the first one.

The sheared chromatin was then incubated with antibodies. For this, 100 μl of sheared chromatin was incubated with 10 μl of pre-washed antibody-coated magnetic beads on a rotating wheel for 2 h at 40 rpm at 4°C. Then, the sample was washed three times with 100 ml of RIPA buffer by centrifugation for 4 min. Finally, the sample was washed with TE buffer in the same way.

To isolate DNA from histones, 100 μl of DNA isolation buffer (20 mM Tris-HCl, pH 7.5, 5 mM EDTA, 20 mM sodium butyrate, 50 mM NaCl, 1% SDS, 50 μg/ml proteinase K) was added to the beads followed by an incubation step at 55°C/15 min and 100°C/15 min. Subsequently, the sample was centrifuged with 14,000 rpm for 5 min at 4°C. The supernatant was then ready for successive qPCR analysis.

### Validation of enriched promoters with ChIP in combination with real-time polymerase chain reaction

To validate the ChIP assays, we performed quantitative real-time PCRs. For this, primers for enriched sequences, including 1 kb upstream and downstream from the peak center, were generated. Primer sequences were published in our previous work [12]. Additionally, we performed ChIP assays with chip-grade antibodies against protamine-1 (catalog no. MAb-001, Briar Patch Biosciences, Livermore, CA, USA). Two microliters of immunoprecipitation sample was used for the PCR reaction with 10 pmol/μl of each primer, 12.5 μl iQTM SYBR Green Supermix (catalog no.170-8882, Bio-Rad, Hercules, CA, USA) in 23 μl of the total volume. For the PCR, we choose the following conditions: an initial step of 3 min at 95°C, followed by 40 cycles of 30 s at 95°C, 30 s at 58°C to 62°C, and 1 min at 72°C. Furthermore, PCR products were checked by sequencing (Scientific Research and Development GmbH).

Calculations for graphs were processed as follows: A threshold was set in the log-linear range for all reactions. The cycle number at which one of the individual PCR reactions reaches this fixed threshold is defined as the Ct-value. To see the enrichment of the immunoprecipitated sample compared to input material, the following calculation was made: ΔCt = Ct (input) - Ct (immunoprecipitated sample) and % total = 2^ΔCt^ × 10 (according to 10% input chromatin of total immunoprecipitated chromatin).

### Pyrosequencing

To quantify the methylation of single CpGs, 20 ng of bisulphate-treated DNA was amplified by PCR with specific primers. These primers were part of the ready-to-use assays provided from Qiagen, Hilden, Germany. We used the following assays: Hs_NSD1_01 (PM00022988), Hs_USP9X_01 (PM00033257), Hs_POU2F1_05 (PM00089103), Hs_RUVBL1_01 (PM00109172), Hs_AFF4_02 (PM00115024), Hs_LRP5_03 (PM00154007), Hs_NCOA6_01 (PM00198044), and Hs_EP300_02 (PM00199731). Additionally, we designed another primer pair for AFF4 to investigate more CpGs in that CpG island. Furthermore, two primer pairs for the protamine-1 promoter region were custom-designed. These three primers were designed with Qiagen (Qiagen, Hilden, Germany) ‘PyroMark Assay Design’ software (Version 2.0.1.15) and are shown in Table 4.

**Table 4:** **Primers designed for pyrosequencing**

**Gene**	**Primers**		**Sequence to analyze**	**PCR product size (bp)**
PRM_1	F	5′-GGG GTG GTT AGG GAT ATG T-3′	ATTTYGTGYGAGYGTTYGTTTAGGTTTTTTAYGYGGTATYGGATTATGGTGTTGGGAGATTTGGTGG	335
	R	Bio5′-CAC CAA ATC TCC CAA CAC CAT-3′		
	S	5′-ATG TAA TTG TTG TTT GTA T-3′		
PRM_2	F	Bio5′-GGG GTG GTT AGG GAT ATG T-3′	ACCRAATCCACAAACRACAACATCRCTCCTACAAAAAACRCAAAAAACRCTCCTACAAACACC	182
	R	5′-AAA AAC CCA TAA CCA ATC TCA CTA TAA-3′		
	S	5′-CAC TAC TCT CCA AAA AAA CTA C-3′		
AFF4	F	5′-GGG TTA GGG TTG GGA TAG TTG A-3′	TTYGTTTYGTTYGTTGGYGGYGGYGAYGGTAGTTGGATTTTTGTAGTTAGGGT	229
	R	Bio5′-AAA CCC CCC CCC CCT ACT-3′		
	S	5′-GAA GAT TTG GTA TTA GGA TT-3′		
H19_4	F	5′-GGGGGTTTTTGTATAGTATATGGGT-3′	AATYGGAAGTGGTYGYGYGGYGGTAGTGTAGGTTTATATATTATAGTT	242
	R	Bio5′-ACTCCTATAAATATCCTATTCCCAAAT-3′		
	S	5′-GGAATTGGTTGTAGTTGTGG-3′		
NSD1m1	F	5′-AGTTGAGGATTTAGTAGGTTTGTATTTAG-3′	GTGTAGTYGTTTYGGTYGGTTYGTTTGYGGTTTGYGTATYGTYGTTGTAAAGGTTT	173
	R	Bio5′-TACCCCCCCCTTCCTAACT-3′		
	S	5′-GGAAGGAGAGTTTTGGG-3′		

Table shows sequence to analyze and size of the PCR amplicons. F: forward primer; R: reverse primer; S: sequencing primer; Bio: biotinylation.

The PCR reaction contained 12.5 μl of PyroMark PCR Master Mix (Qiagen, Hilden, Germany), 2.5 μl of CoralLoad (Qiagen, Hilden, Germany), and 2.5 μl of 0.2 μM primer mix, together with DNA diluted with ddH_2_O in to a final volume of 25 μl. The following conditions were used for PCR: 95°C for 15 min followed by 45 cycles at 95°C for 30 s, 56°C for 30 s, and 72°C for 30 s and a final extension at 72°C for 10 min. The PCR products were pyrosequenced in Qiagen PyroMark Q24. We used the standard protocol and the required chemicals supplied by Qiagen. The sequencing primers were part of the previously described assays. The received data was analyzed by Qiagen Q24 software (Version 2.0.6) (Qiagen, Hilden, Germany).

### Immunostaining of mouse spermatozoa with anti-H4K12ac and anti-5mC antibodies

Spermatozoa were obtained from C57Bl/6mice at the age of 4 to 6 months. Sperm from the caudae epididymidae was extracted in to 1 ml of TBS followed by centrifugation for 8 min at 3,000 rpm. The pellet was re-suspended with PBS and diluted in distilled water. Five-microliter droplets were smeared onto a glass slide and dried for 2 h at room temperature (RT). Smears were covered by a freshly prepared decondensing mix (25 mM DTT, 0.2% Triton X-100, and 200 IU heparin/ml) and incubated at 37°C under 5% CO_2_ for 12 to 15 min according to the state of the decondensation, which was monitored under a light microscope. The decondensation was stopped by the fixation of sperm in 3.7% paraformaldehyde for 20 min followed by washing in PBS. The prepared slides were blocked with 10% goat serum in PBS for 2 h at RT, washed and covered by a mixture of anti-5mC (BI-MECY-0500 Eurogentec, Cologne, Germany) and anti-H4K12ac antibodies (ab61238, Abcam, Cambridge, UK) (both antibody in the dilution of 1:500 in PBS) and incubated for 2 h at RT. After the washing steps, 10 times in PBS, secondary antibodies with fluorescein thiocyanate (FITC) conjugate (goat polyclonal secondary antibody to rabbit IgG - H&L (FITC), ab6717, Abcam, Cambridge, UK) and Alexa Fluor® 568 Goat Anti-Mouse IgG (H + L) A-11004, Molecular Probes, Invitrogen, Karlsruhe, Germany) were diluted 1:1,000 in PBS and slides were incubated for 1 h. After washing, slides were mounted with VECTASHIELD mounting medium for fluorescence with DAPI (H-300, Vector Laboratories, Inc., Burlingame, CA, USA).

### Mouse *in vitro* fertilization and immunostaining of mouse pronuclei

C57Bl/6 mice were obtained from a breeding colony of the Laboratory of Reproduction, Faculty of Science, Charles University in Prague. All animal procedures were carried out in strict accordance within the law of the Czech Republic, paragraph 17 no. 246/1992, and Animal Scientific Procedures, paragraph 11, no. 207/2004, and the Local Ethics Committee of the Faculty of Science of Charles University in Prague specifically approved this study in accordance with accreditation no. 24773/2008-10001.

While male mice were within a reproductive age of 10 to 12 weeks, the female mice used for hormonal stimulation were 21 days old. For the *in vitro* fertilization, the isolated eggs from the oviduct of juvenile hormonally stimulated females (strain C57Bl/6) were added to capacitated sperm gained from the distal region of cauda epididymis for fertilization. Zona pellucida was removed by Tyrodes solution (catalog no. T1788, Sigma-Aldrich, Seelze, Germany), and the eggs were fixed for 20 min in 2.5% paraformadehyde. TritonX-100 (0.1%) in PBS was added for 10 min to wash and permeabilize the eggs. After blocking with goat serum, embryos and eggs were washed three times with 0.05% Tween in PBS incubated with primary antibodies. A mixture of anti-5hmc (39769, Active Motif, Rixensart, Belgium) and anti-5mC (BI-MECY-0500 Eurogentec, Cologne, Germany) antibodies (dilution 1:1000 both) was applied. Other embryos were stained with anti-H4K12ac (ab61238, Abcam, Cambridge, UK) and anti-5mC (BI-MECY-0500 Eurogentec, Cologne, Germany) (dilution 1:1,000 in 1% goat serum in PBS). Eggs and embryos were incubated with primary antibodies for 3 h in RT and then washed three times in 0.05% Tween in PBS. Secondary antibodies Alexa Fluor 488 (FITC) goat anti-rabbit IgG (A-11008, Molecular Probes, Invitrogen, Karlsruhe, Germany) and Alexa Fluor 568 goat anti-mouse IgG, (A-11004, Molecular Probes, Invitrogen, Karlsruhe, Germany) or secondary antibodies anti-rabbit IgG (FITC), (ab6717, Abcam, Cambridge, UK) and Alexa Fluor 568 Goat anti-Mouse IgG (A-11004, Molecular Probes, Invitrogen, Karlsruhe, Germany) were diluted 1:1,000 in PBS. Incubation with secondary antibodies was held for 1 h, and eggs were then washed three times in 0.05% Tween in PBS and mounted on slides with VECTASHIELD mounting medium for fluorescence (H-100, Vector Laboratories, Inc., Burlingame, CA, USA). Samples were examined with a Leica DM IRE2 (Leica, Heidelberg, Germany) high-speed confocal/two photon system for Live Cell Imaging.

### Statistics

The Mann-Whitney *U*-test was applied in order to compare the enrichment of μChIP assay between the groups of fertile and infertile patients. The same Mann-Whitney *U*-test was performed for the comparison of methylation levels in promoters between fertile donors and subfertile patients as values were not normally distributed. Correlations of clinical parameters with cumulative methylation in each investigated promoter were tested by non-parametric Spearman’s rho correlation. Differences were considered significant when *P* < 0.05. Statistical calculations were performed using GraphPad Prism, version 5.02 for Windows (GraphPad Software, San Diego, CA, USA).

## Additional files

Additional file 1:
**Methylation analysis of the selected H4K12ac interacting promoters POU1F2, NCOA6, LRP5, EP300, USPX9, and RUVBL1.** A: The enrichment of binding sites for selected H4K12ac-associted promoters in sperm chromatin of fertile and subfertile men (ChIP-chip assay Hg18 NimblGene). Each feature on the array had a corresponding scaled log2 ratio that was calculated from the input signal Cy3 for the total of chromatin and IP probe - Cy5, which were co-hybridized to the array. The log2 ratio was computed and scaled to the center the ratio data around zero. Scaling was performed by subtracting the bi-weight mean of the log2 ratio values for all features on the array from each log2 ratio value. The binding sites for H4K12ac to sperm chromatin were detected by searching for four or more oligo probes whose signals were above the specific cut off values, ranging from 90% to 15%, using a 500-bp sliding window. The ratio data were randomized 20 times, and each peak was assigned a false discovery rate (FDR) score based on the randomization. The lower the FDR score, the more likely the peak corresponded to a H4K12ac binding site. Data are visualized using SignalMap browser (NimbleGen). B: Genomic position of analyzed CpGs within CpG islands of selected genes - pyrosequencing. C: Methylation levels of each investigated CpG in sperm DNA of fertile and subfertile patients. Additional file 2:
**Figure demonstrating immunofluorescent labeling of nuclei of parthenogenetically activated oocytes.** Nuclei stained with antibody anti-H4K12ac (green) (A), DAPI (blue) (B), merged (C), merged with DIC (D); antibodies anti-H4K12ac (E), anti-5mC (F), merged (G), merged with DIC (H); and antibodies anti-5hmC (I), anti-5mC (J), merged (K), merged with DIC (L).

## Abbreviations

AFF4: AF4/FMR2 family member 4
AGPAT6: glycerol-3-phosphate acyltransferase 4 precursor
AXIN1: axis inhibition protein 1
BRDT: bromodomain testis-specific protein
CBX8: chromobox protein homologue 8
ChIP: chromatin immunoprecipitation assay
ChIP-chip: chromatin immunoprecipitation assay in combination with microarray
CTCF: CCCTC-binding factor
Cy3: cyanine 3 conjugate
D2: day 2, four-cell embryo
D3: day 3, eight-cell embryo
D5: day 5, blastocyst
DAPI: 4′, 6-diamidino-2-phenylindole fluorescent nucleic acids stain
DIC: differential interference contrast
DVL1: segment polarity protein disheveled homologue DVL-1: EP300
FITC: fluorescein thiocyanate
H3K27me3: histone H3 trimethylated at lysine 27
H3K4me3: histone H3 trimethylated at lysine 4
H3K9ac: histone H3 acethylated at lysine 9
H4K12ac: histone H4 acetylated at lysine 12
HDAC6: histone deacetylase 6
HIST1H2BC: histone H2B type 1-C/E/F/G/IHSF2BP heat shock factor 2-binding protein
IFT172: intraflagellar transport protein 172 homologue
ITCH: myosin light chain 3
KDM2A: lysine-specific demethylase 2A
MAPK8IP3: C-jun-amino-terminal kinase-interacting protein 3
MAST2: microtubule-associated serine/threonine-protein kinase 2
MI: metaphase I oocyte
MII: metaphase II oocyte
MLH3: DNA mismatch repair protein Mlh3
NCOA6: nuclear receptor coactivator 6 NSD1:histone-lysine *N*-methyltransferase
PEX9: peroxisomal membrane protein 9
PHF7: testis-specific PHD finger protein-7
PSMC4: 26S protease regulatory subunit 6B
RUVBL1: U6 spliceosomal RNA
STAG3: cohesin subunit SA-3 (stromal antigen 3)
TCFL5: transcription factor-like 5 protein
TRIP13: thyroid receptor-interacting protein 13
TSS: transcription star site
USP9X: probable ubiquitin carboxyl-terminal hydrolase FAF-X
WHO: World Health Organization
ZEB1: zinc finger E-box-binding homeobox 1
