# Supplementary figures and images for: Methylation analysis of histone H4K12ac-associated promoters in sperm of healthy donors and subfertile patients
Source: Clin Epigenetics. 2015 Mar 19;7(1):31. doi: 10.1186/s13148-015-0058-4 (PMC4372182; doi:10.1186/s13148-015-0058-4)

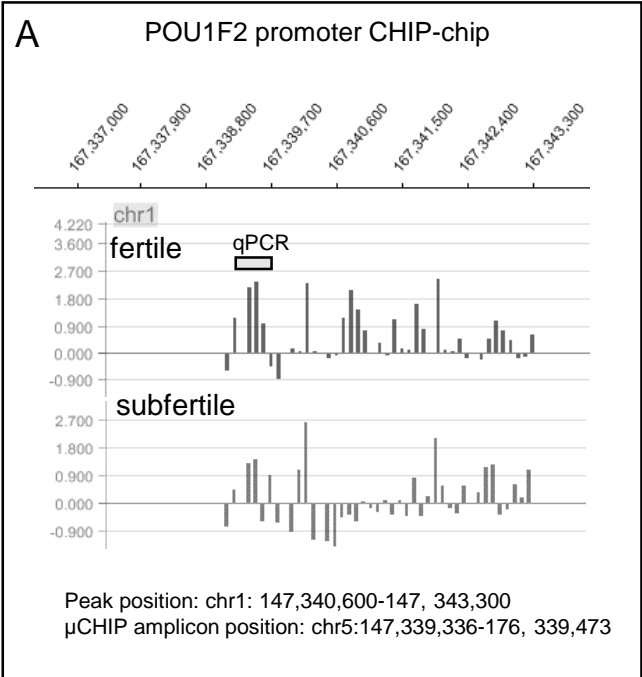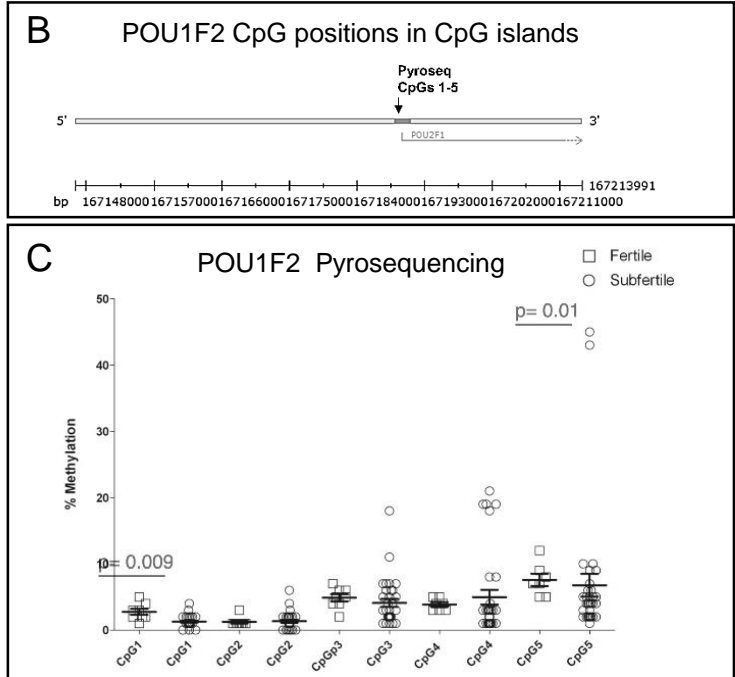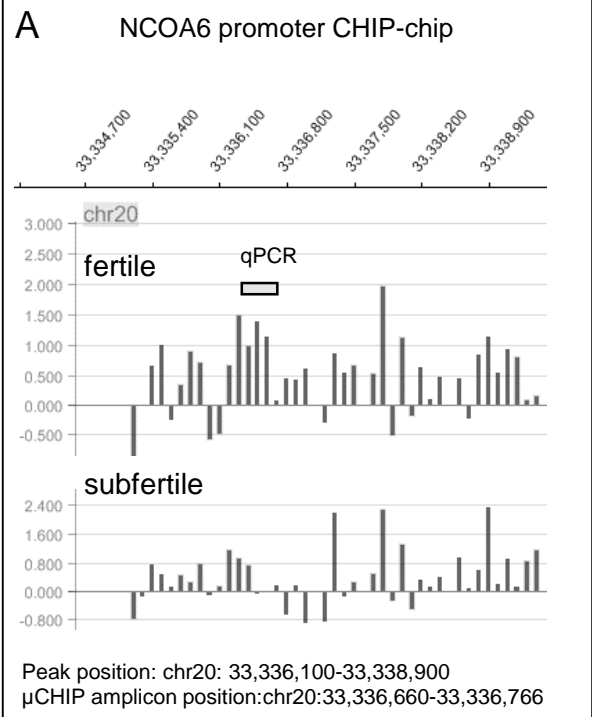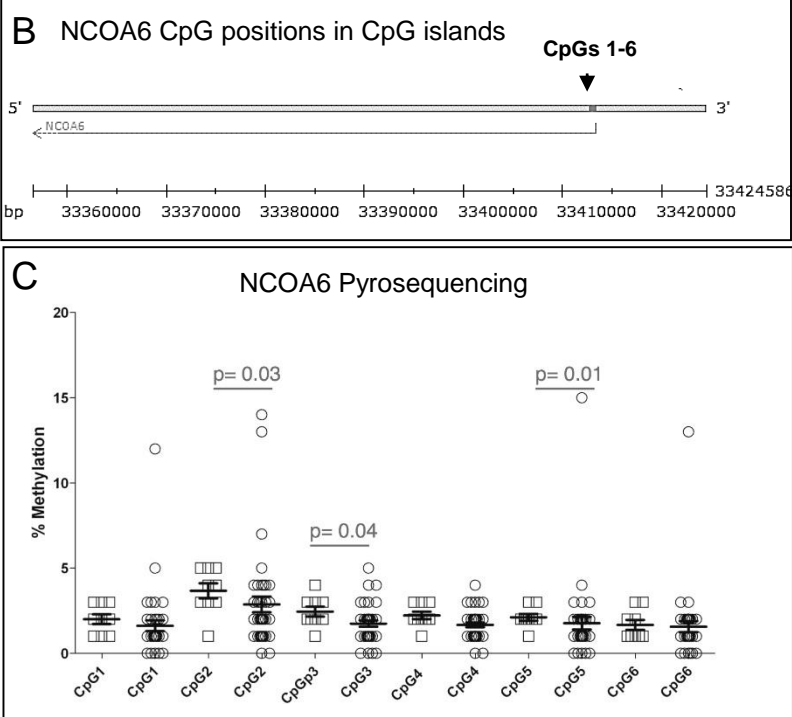

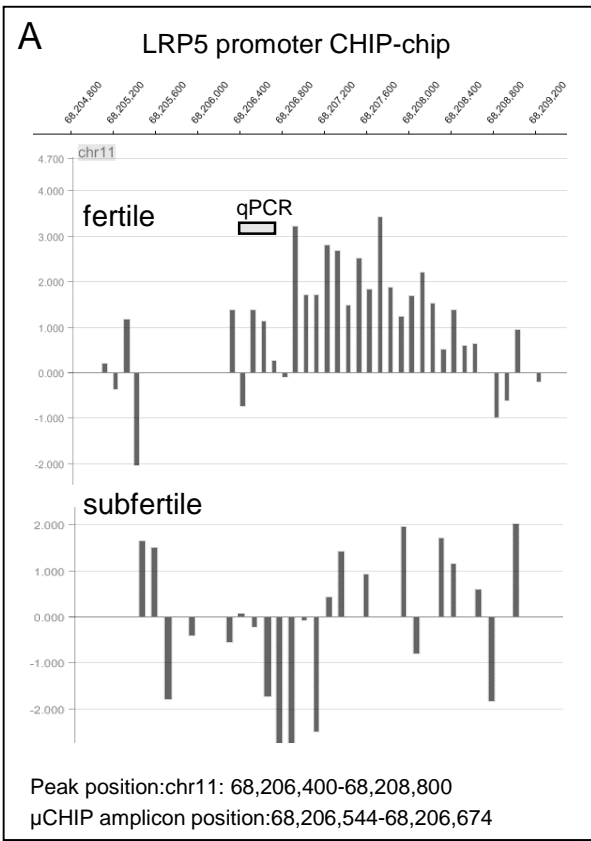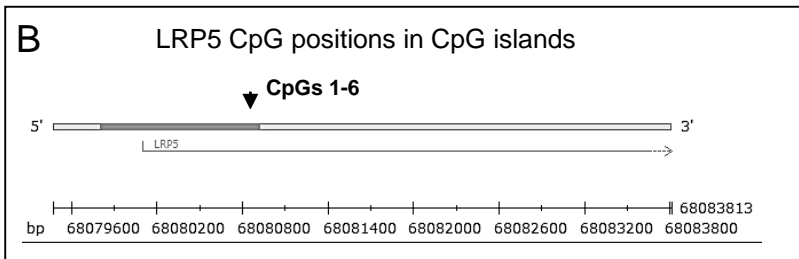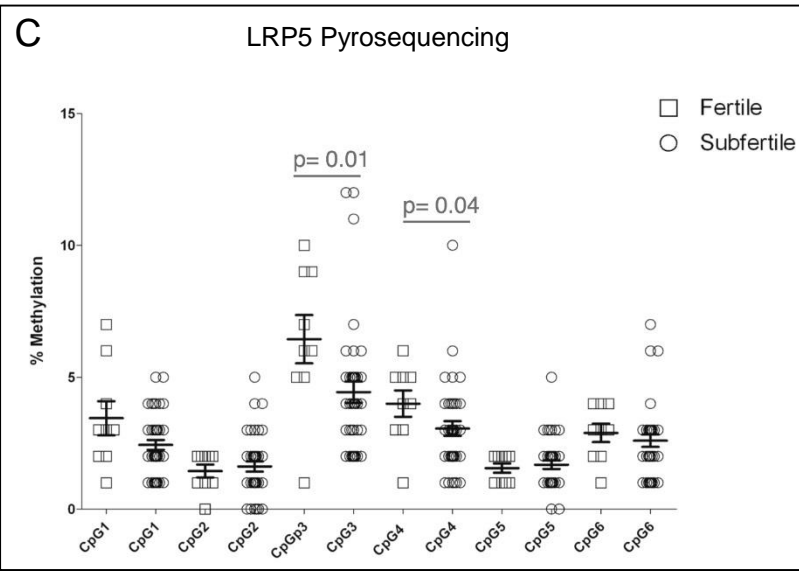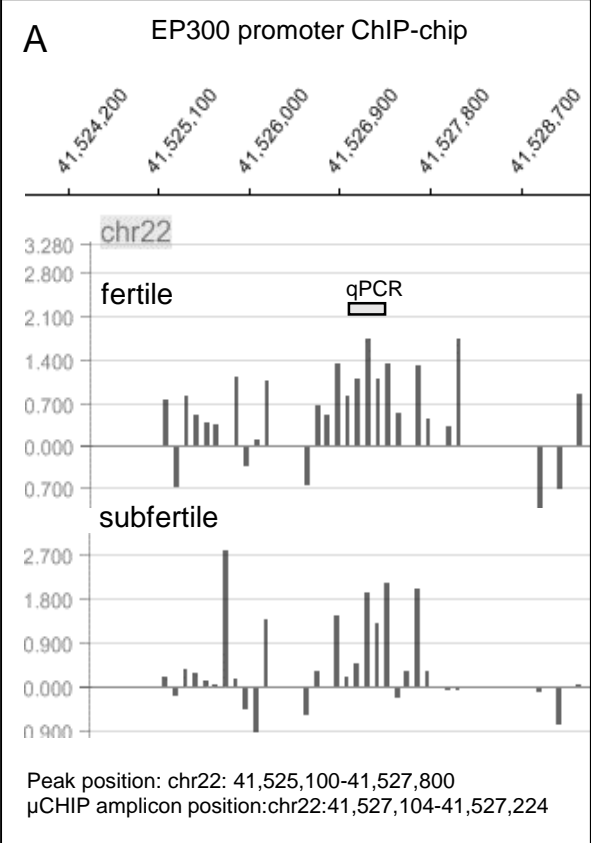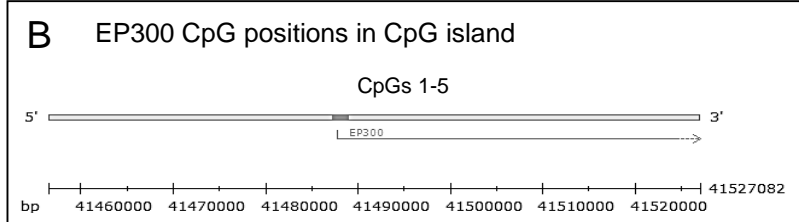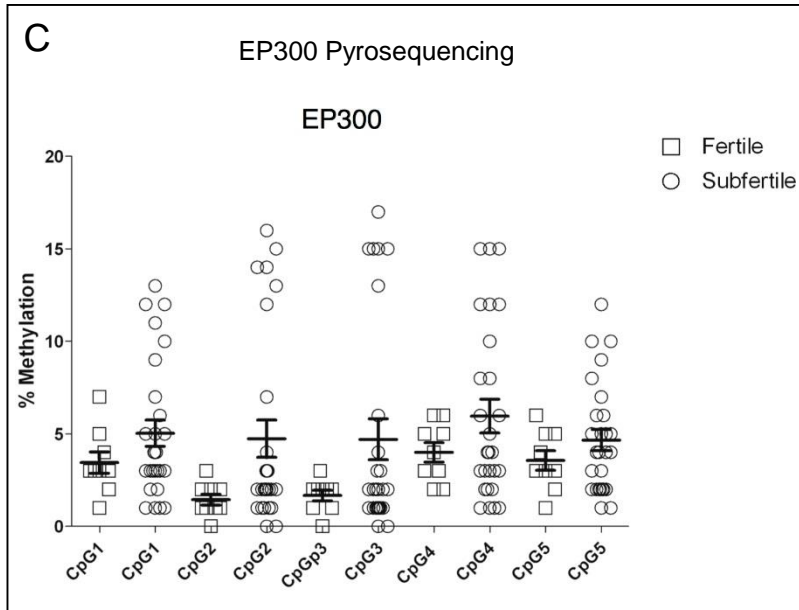

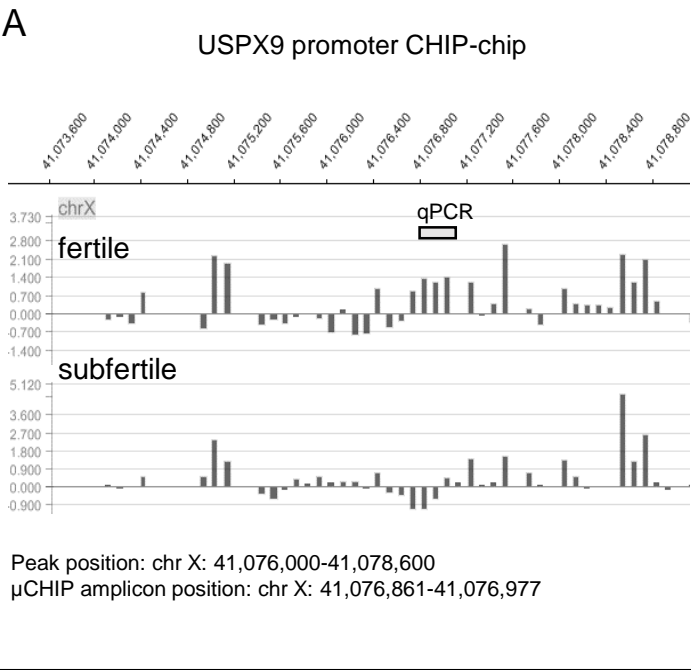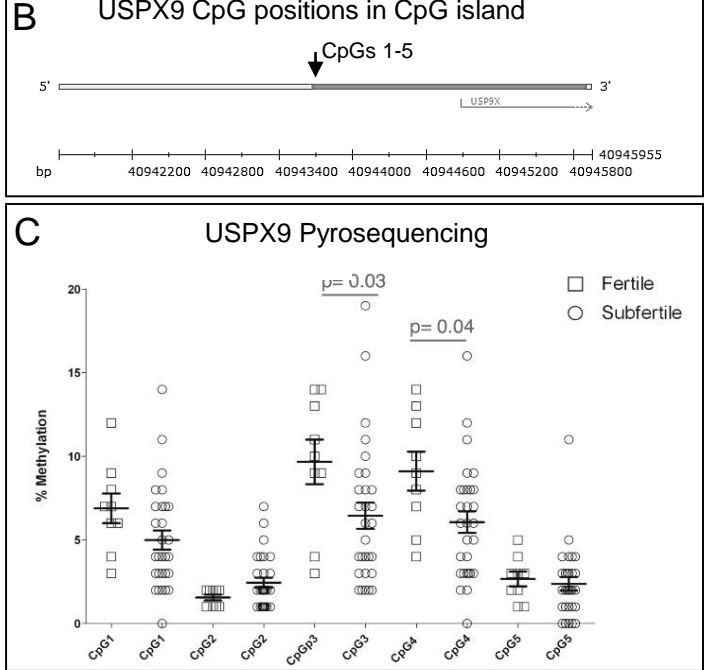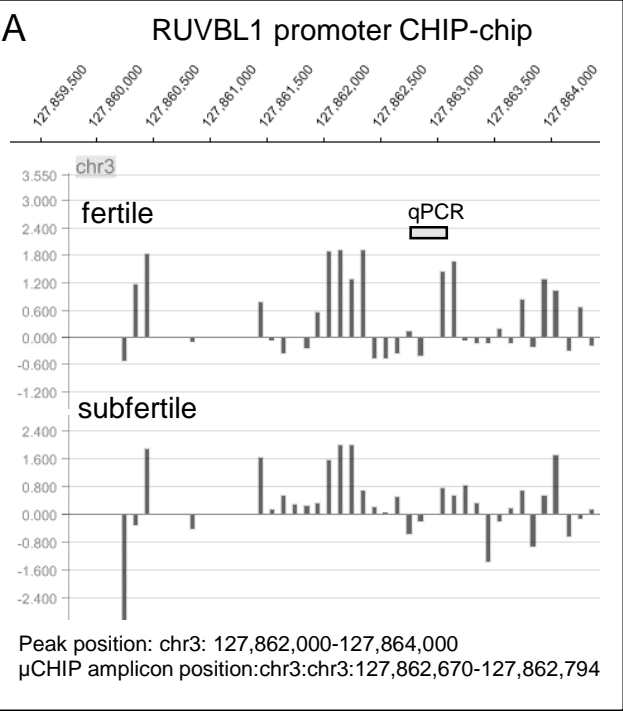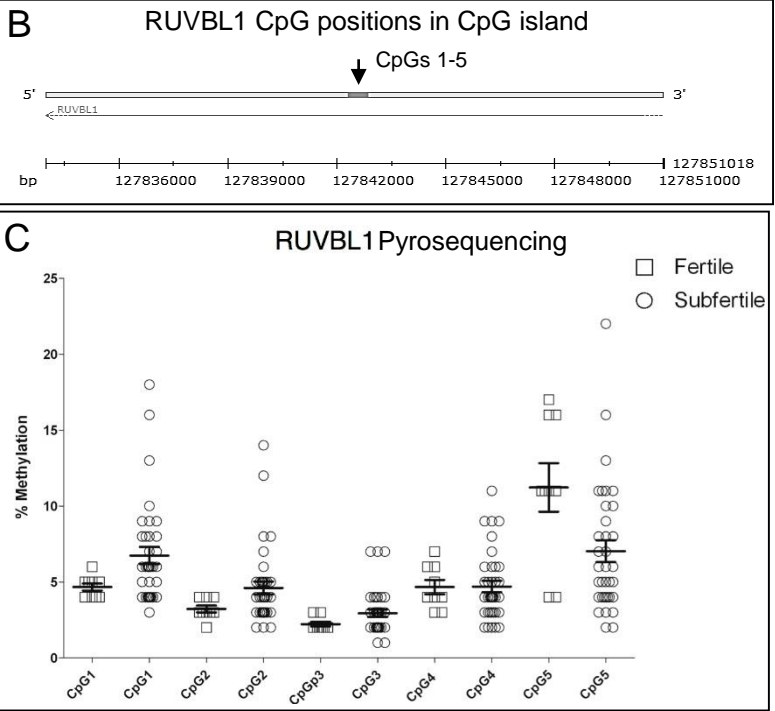

Supplement: Additional file 1: — Methylation analysis of the selected H4K12ac interacting promoters POU1F2, NCOA6, LRP5, EP300, USPX9, and RUVBL1. A: The enrichment of binding sites for selected H4K12ac-associted promoters in sperm chromatin of fertile and subfertile men (ChIP-chip assay Hg18 NimblGene). Each feature on the array had a corresponding scaled log2 ratio that was calculated from the input signal Cy3 for the total of chromatin and IP probe - Cy5, which were co-hybridized to the array. The log2 ratio was computed and scaled to the center the ratio data around zero. Scaling was performed by subtracting the bi-weight mean of the log2 ratio values for all features on the array from each log2 ratio value. The binding sites for H4K12ac to sperm chromatin were detected by searching for four or more oligo probes whose signals were above the specific cut off values, ranging from 90% to 15%, using a 500-bp sliding window. The ratio data were randomized 20 times, and each peak was assigned a false discovery rate (FDR) score based on the randomization. The lower the FDR score, the more likely the peak corresponded to a H4K12ac binding site. Data are visualized using SignalMap browser (NimbleGen). B: Genomic position of analyzed CpGs within CpG islands of selected genes - pyrosequencing. C: Methylation levels of each investigated CpG in sperm DNA of fertile and subfertile patients. [file 13148_2015_58_MOESM1_ESM.pdf]

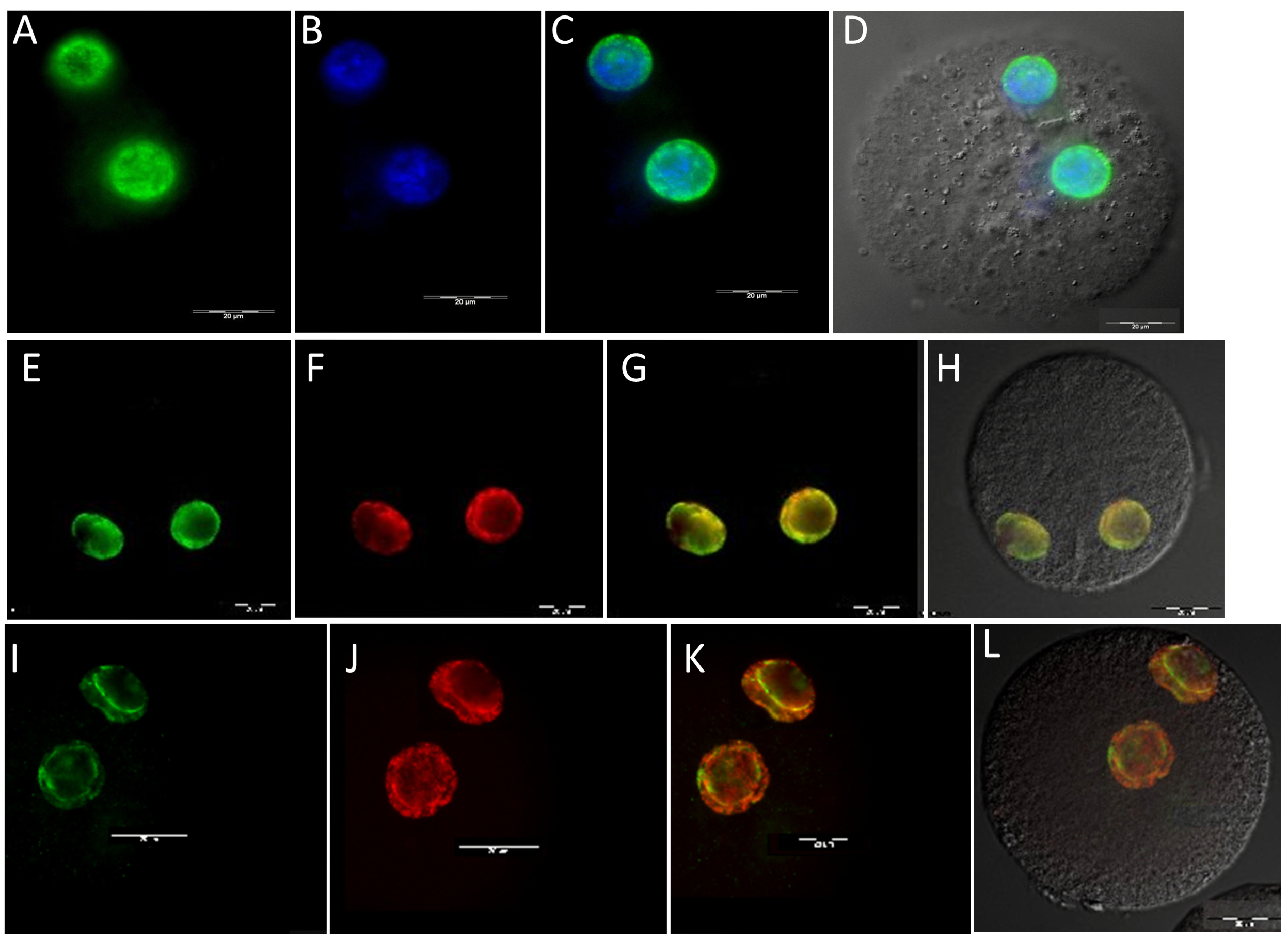

Supplement: Additional file 2: — Figure demonstrating immunofluorescent labeling of nuclei of parthenogenetically activated oocytes. Nuclei stained with antibody anti-H4K12ac (green) (A), DAPI (blue) (B), merged (C), merged with DIC (D); antibodies anti-H4K12ac (E), anti-5mC (F), merged (G), merged with DIC (H); and antibodies anti-5hmC (I), anti-5mC (J), merged (K), merged with DIC (L). [file 13148_2015_58_MOESM2_ESM.tiff]
